# Colorimetric Comparison of Internal Bleaching with and without Removing Mineral Trioxide Aggregate (MTA) on Induced Coronal Tooth Discoloration by MTA

**DOI:** 10.1155/2021/8319986

**Published:** 2021-09-27

**Authors:** Sedigheh Khedmat, Elham Ahmadi, Naghmeh Meraji, Zahra Farhang Fallah

**Affiliations:** ^1^Dental Research Center, Dentistry Research Institute, Tehran University of Medical Sciences, Tehran, Iran; ^2^Department of Endodontics, School of Dentistry, Tehran University of Medical Sciences, Tehran, Iran; ^3^Department of Operative Dentistry, School of Dentistry, Tehran University of Medical Sciences, Tehran, Iran; ^4^Board Certified Endodontist, Private Practice, Tehran, Iran; ^5^General Dentist, Tehran, Iran

## Abstract

*Objective*. This study aimed to colorimetric comparison of internal bleaching with and without removing mineral trioxide aggregate (MTA) on induced coronal tooth discoloration by MTA cement. In this experimental study, twenty human teeth were prepared. An OrthoMTA barrier was placed 1 mm below the CEJ. The teeth were restored with composite resin and were placed in the aging accelerator machine. Then, the specimens were divided into two groups (*n* = 10); in group A, part of the OrthoMTA was removed and the glass ionomer was placed on the OrthoMTA, and in group B, the OrthoMTA remained intact. Internal bleaching was performed 5 times in 6-day intervals using 37% carbamide peroxide gel. Color determination was performed in 5 stages: baseline, after OrthoMTA discoloration, before OrthoMTA removal, after OrthoMTA removal, and after bleaching treatment sessions. In group A, 8 specimens reached to ∆*E* < 3.3 after 2 times internal bleaching treatment, and in group B, 5 specimens reached to ∆*E* < 3.3 with almost 3 bleaching sessions (*p* > 0.05). Additionally, 5 specimens reached to the initial color (baseline) after bleaching treatment, 4 specimens in group A and 1 specimen in group B. After OrthoMTA removal, 2 specimens in group A reached to ∆*E* < 3.3. There was no significant difference between groups with or without OrthoMTA removal (*p*=0.06). Although, the specimens with OrthoMTA removal required fewer bleaching treatment sessions, and the mean value of ∆*E* was lower in this group.

## 1. Introduction

Mineral trioxide aggregate (MTA) has been widely used in different endodontic treatments including regenerative endodontic procedures (REPs) [1, 2]. MTA is a mixture of tricalcium silicate, dicalcium silicate, tricalcium aluminate, tetracalcium aluminoferrite, and bismuth oxide [3, 4]. This cement has high biocompatibility and low cytotoxicity [5, 6]. One of the important drawbacks of MTA is the crown discoloration in anterior teeth [7–11]. This problem resulted to 31.6–57% patient dissatisfaction [12].

The first commercial formulation was gray MTA (GMTA) and had the high potential to cause tooth discoloration. Metal oxides such as Al_2_O_3_, MgO, and FeO present in GMTA were assumed to be the main causes of discoloration. White MTA (WMTA) includes fewer metal oxides such as Al_2_O_3_, MgO, and FeO and was developed to overcome this disadvantage, but yet, WMTA has been shown to cause tooth discoloration [11].

Bismuth oxide, the radiopacifier of both types of MTA, has been suggested as the cause of tooth discoloration [10, 13, 14]. It has been stated that oxidation of this material destabilizes the oxygen in its formulation, which reacts with carbon dioxide and produces bismuth carbonate that causes discoloration [11, 15]. Another theory is the interaction of bismuth oxide with dentin collagen [4, 10, 16]. In addition, the interaction of MTA slurry with blood during its hydration may contribute to discoloration [9, 17, 18]. Blood in the root canal adjacent to the MTA influences tooth discoloration in REPs [9].

Internal bleaching is a conventional method in the treatment of teeth with crown discoloration [1, 19]. According to the research studies, placing 2-3 mm of an impermeable base such as resin-modified glass ionomer as a cervical barrier is recommended before internal bleaching to prevent cervical root resorption [20]. Some suggested that bleaching materials could be applied on the MTA present in the cervical region of teeth with discoloration due to its proper sealing ability [19]. On the other hand, applying the bleaching materials over the MTA may lead to the weakening of MTA's structure caused by the high acidity of the bleaching agents [20].

In several studies, complete removal of MTA has recommended. They stated partial MTA removal may destruct the sealing obtained by MTA [8, 21, 22]. Two studies reported an extensive improvement in the color of the tooth by completely removing discolored MTA after REPs [8, 21]. Another study concluded that partial removal of MTA and replacing it with glass ionomer prevented the adverse effects of bleaching agents on MTA. On the other hand, the calcium released from the remaining MTA, and its alkaline pH protects the root from external resorption [23].

The aim of this ex vivo study was the colorimetric comparison of internal bleaching with and without removing mineral trioxide aggregate (MTA) on induced coronal tooth discoloration by MTA.

## 2. Materials and Methods

The study was approved by the Ethics Committee of Tehran University of Medical Sciences, Tehran, Iran (IR.TUMS.DENTISTRY.REC.1397.129).

Twenty human permanent maxillary central teeth that were extracted due to periodontal problems were chosen. The teeth were caries-free and had no coronal restoration, crack, or fractures. The soft tissues were removed from the teeth by polishing with pumice paste and water. For disinfection, they were immersed in chloramine *T* 0/5% solution for one week and then stored in the normal saline solution until use. Selected teeth were approximately 15 ± 1 mm long totally.

### 2.1. Blood Collection

Fresh human blood was collected from a volunteer member of the research group by an expert individual according to the Helsinki ethical principles for medical research relating to human subjects [24].

### 2.2. Preparation of Specimens

The apical part of each root was cut perpendicular to its long axis with a high-speed diamond tapered fissure bur #018 (Jota, Rüthi, Switzerland) with a continuous spray of water until 7 mm of root remained. Endodontic access cavities were prepared, and the cleaning and shaping of root canals were done using #1–6 Gates-Glidden drills (Mani, Tochigi, Japan) and irrigated with distilled water.

OrthoMTA (BioMTA, Seoul, Korea) was mixed with distilled water according to the manufacturer's instruction. A thickness of 3 mm of the MTA was placed in the coronal area of the root canal 1 mm below the CEJ. Periapical radiographic (Kodak, Marne-la-Vallée, France) was taken to confirm sufficient and uniform MTA content and thickness. Then, a wet cotton pellet was placed on the MTA, and the teeth were restored with Coltosol (Asia Chemi Teb Co., Tehran, Iran). Customized foam saturated with human fresh blood was inserted into the root canal through the apical opening up to the MTA barrier, and the root ends were sealed with a glass ionomer (AHL, Tonbridge, United Kingdom).

### 2.3. Experimental Setup

**Figure 1:**
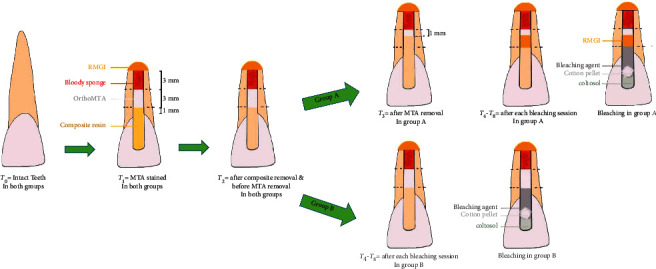
Schematic illustrations of the procedure of this study.

The teeth were incubated at 37°C and fully saturated humidity for 24 hours. After 24 hours, the temporary restoration and wet cotton pellet were removed and the setting of the MTA was checked; then, the crown of the specimens was restored with composite resin material (Estelite ∑, Tokuyama Dental Corp, Tokyo, Japan, shade *A*_1_). The teeth were put in the accelerating artificial aging (AAA) machine for 75 hours at 37°C with full saturation moisture and UV irradiated to simulate the oral cavity situation equal to 3 months in the clinical conditions. Then, the specimens were randomly divided into two experimental subgroups (*n* = 10). First, composite restoration was eliminated from all specimens, and then, in group A, 2 mm of the MTA was removed using an ultrasonic (NSK, Tochigi, Japan) device under a microscope (Carl Zeiss dental microscope, ×10 magnification), and a glass ionomer (AHL, Tonbridge, United Kingdom) was placed on the MTA as a barrier 1 mm below the CEJ. In group B, the MTA remained intact. The bleaching agent containing 37% carbamide peroxide gel (FGM, Whiteness superendo, Joinville, SC Brazil) was inserted into the access cavities of all specimens and covered with a small cotton pellet, and the cavities were sealed with Coltosol. The bleaching procedure was performed in all teeth 5 times at 6-day intervals. Color changes were measured after every bleaching session (Figure 1).

### 2.4. Color Assessment

The specimens were mounted in silicon blocks (Coltene, Altstatten, Switzerland) for reproducible tooth positioning. Tooth color measurements were performed under similar light conditions and in a dark room using a spectrophotometer (VITA Zahnfabrik, Bad Sackingen. Germany). Before each color measurement, device calibration was performed according to manufacturer's instruction.

The area used to determine the color of teeth was the part between 1/3 cervical and 1/3 middle that two-thirds of its height was in the cervical and one-third was in the middle-third of the tooth crown. The light source was matched to the surface of the mentioned area, and the color determination was performed.

Tooth color measurements were done at 5 steps:

1. *T*_0_ = intact (in both groups)
2. *T*_1_ = MTA-stained (in both groups)
3. *T*_2_ = after composite removal and before MTA removal (in group A)
4. *T*_3_ = after MTA removal (in group A)
5. *T*_4_–*T*_8_ = after each bleaching session (in both groups)

The mean value of 3 times color measurements was considered at each step.

The color change (Δ*E*^*∗*^) of each specimen was calculated using the following equation [25].

(1) $$\begin{array}{c} \Delta E={\left[{\left(\Delta L^{*}\right)}^{2}+{\left(\Delta a^{*}\right)}^{2}+{\left(\Delta b^{*}\right)}^{2}\right]}^{0.5}. \end{array}$$

In color assessment by a spectrophotometer, three main parameters are determined that each of them represents a specific spectrum of colors.

The *L*^*∗*^ (lightness) parameter represents brightness, which ranges from black (0) to white (100). Parameter *L*^*∗*^ indicates the value. The parameter *a*^*∗*^ represents the greenness (negative value) and redness (positive value) spectra, and *b*^*∗*^ represents the blueness (negative value) and yellowness (positive value) spectra. Parameters *a*^*∗*^ and *b*^*∗*^ indicate the chroma.

### 2.5. Statistical Methods

Repetitive analysis of variance (post-Sidak test) was used to assess the effect of MTA removal on tooth color changes during bleaching treatment. An independent *t*-test was used to compare the bleaching frequency between the two groups. The fisher test was used to compare the percentage of color correction between the two groups. The statistical significance limit was less than 0.05%.

## 3. Result

The color assessment was done in 5 stages (Table 1), and ∆*E*, ∆*L*, and ∆*b* were calculated for both groups (Table 2).

According to Δ*E*_1_ (intact and MTA stained), all samples had a color change more than 3.3 after the process in the artificial accelerated aging device. The specimens were divided into two subgroups by stratified randomization. The mean value of Δ*E*_1_ in group A was 9.12 ± 5.78 and in group B was 9.67 ± 6.17.

### 3.1. Δ*L*^*∗*^

**Figure 2:**
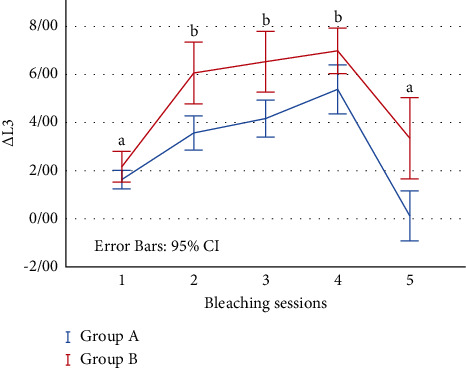
The mean values of ∆*L*_3_ (MTA-stained and bleaching sessions) during bleaching sessions. There is a statistically significant difference between a and b (*p* < 0.05).

In both groups, Δ*L*_3_ (MTA-stained and bleaching sessions) increased during the 1–4 bleaching sessions and the specimens became brighter, but after the fifth session of bleaching, Δ*L*_3_ decreased (Figure 2).

There was no significant difference between two groups after each bleaching session (*p*=0.1). In both groups, Δ*L*_3_ was considerably different between session 1 and sessions 2, 3, and 4, and there was a significant difference between session 5 and sessions 2, 3, and 4 (*p* < 0.05) (Figure 2).

### 3.2. Δ*b*^*∗*^

**Figure 3:**
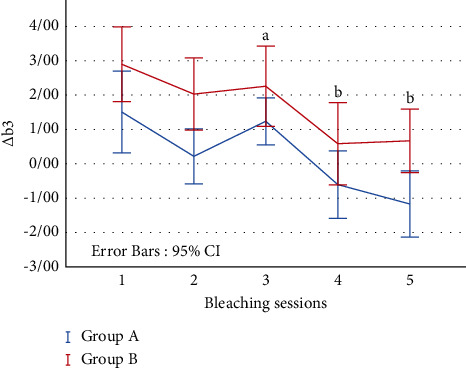
The mean values of ∆*b*_3_ (MTA-stained and bleaching sessions) during bleaching sessions. There is a statistically significant difference between a and b (*p* < 0.05).

Parameter *b*^*∗*^ represents the blueness-yellowness. In both groups, Δ*b*_3_ (MTA-stained and bleaching sessions) first had a decreasing-increasing process during 1–3 sessions and then decreased during 4-5 bleaching sessions (Figure 3).

There was no significant difference between the groups during the bleaching sessions (*p*=0.26), but there was a significant difference (*p* < 0.01) between session 3 and sessions 4 and 5 in both groups (Figure 3).

### 3.3. Δ*E*^*∗*^

In group A, 8 teeth, and in group B, 5 teeth, with 2 and 2.8 average bleaching sessions reached to Δ*E* ˂ 3.3, respectively (*p*=0.42).

**Figure 4:**
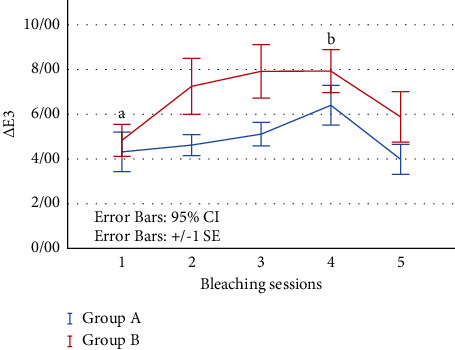
The mean values of ∆*E*_3_ (MTA-stained and bleaching sessions) during bleaching sessions. There is a statistically significant difference between a and b (*p* < 0.05).

According to Figure 4, the mean values of Δ*E*_3_ (MTA-stained and bleaching sessions) during bleaching sessions were higher in group B compared with group A. There was no significant difference between two groups during the bleaching sessions (*p*=0.06).

The amount of Δ*E*_3_ increased during 1–4 bleaching sessions and then decreased after the last session. There was a significant difference between session 1 and session 4 in both groups (*p* < 0.05).

According to Δ*E*_4_ (intact and bleaching sessions), a total of 5 teeth reached ∆*E* ˂ 3.3, in which 4 specimens belonged to group A and 1 specimen belonged to group B.

Based on Δ*E*_2_ (before and after MTA removal) in group A, 2 specimens reached ∆*E* < 3.3 with MTA removal.

## 4. Discussion

In this study, human teeth were used to assess crown discoloration induced by OrthoMTA in the presence of fresh human blood that simulated the clinical situation of regenerative treatment. Artificial accelerated aging (AAA) is used to reconstruct the clinical conditions over time. This method is different from other studies that used only exposure to an aqueous medium [1, 12, 26, 27]. Samples are subjected to a combination of factors such as humidity, thermal changes, UV rays, and visible light [28]. The settings of the device are based on the ISO 7491: 2000 [29], which has a wavelength of 200–800 nm, a xenon lamp, a temperature of 35 ± 5, and a humidity of 100%. In this aging machine, 300 h is equivalent to one year of clinical activity [30, 31], and in the present study, 75 h was considered for 3 months of clinical activity. According to Moazzami et al.' research [32], the maximum discoloration caused by WMTA occurs within 12 weeks, so we chose this time for AAA.

The access cavities were filled with composite during the placement of the samples in the device, so the initial color change created in the samples of the present study was the combined effect of composite and MTA changes, and it is not possible to separate the color changes from each other. Some researchers reported that composite resin did not play a role in the process of discoloration caused by calcium silicate-based cements [10].

To minimize changes in color parameters between two groups, the stratified randomization method was used to divide the samples.

Internal tooth bleaching is a conservative and relatively simple and low-cost method in the treatment of teeth with internal discoloration [33]. External root resorption with a prevalence of 1–12% [34] is a potential complication of this treatment; therefore, placing a cervical barrier is suggested to prevent it.

Various materials such as glass ionomer, MTA, CEM-cement, and resin-modified glass ionomer have been suggested as cervical barriers [35]. A suitable cervical barrier should have the ability to seal properly and be removed easily after bleaching without any negative effects on the bond of restoration [35, 36].

Studies evaluating microleakage [37, 38] and sealing ability [39] have suggested the use of MTA which had placed in the coronal area of the root canal 1 mm below the CEJ as a cervical barrier during internal bleaching. In addition, easier removal of MTA in comparison with other cervical barriers makes it a good choice [38, 40], although there is controversy with some studies that believe MTA removal is harder [41].

A comparison of the WMTA and GMTA by X-ray analysis showed that GMTA has more amount of Al_2_O_3_, MgO, and FeO in its composition that can be the reason for color difference [42]. It seems that significant decrease in the amount of FeO in WMTA is the main reason of less color change by use of WMTA in comparison with GMTA [43].

There is a concern about the adverse effects of bleaching agents on the characteristics of cervical barriers and their chemical and mechanical properties [35]. Changes in the microhardness and surface roughness [39] and discoloration of MTA after contact with bleaching agents [20] have been reported.

Researchers stated only MTA removal in the discolored teeth resulted in a significant improvement in the color of the tooth [8]. Belobrov and Parashos [21] presented a case that showed dentin barrier formation after pulpotomy treatment with MTA, and a considerable improvement in the tooth color was achieved after complete MTA removal. D'Mello and Moloney [23] reported that removing 1 mm of 3 mm of MTA in teeth and replacing it with a glass ionomer prevented the adverse effects of bleaching agents on MTA. On the other hand, the calcium released from the remaining MTA and its alkaline pH protects the root from external resorption. Some studies have reported that complete removal of calcium silicate cements and replacement with other cervical barriers may increase the risk of distracting the seal attained by these cements [8, 21, 22].

Therefore, the present study, in group A, was similar to the study of D'Mello and Moloney [23], 2 mm of the 3 mm MTA was removed and replaced with resin-modified glass ionomer (RMGI).

Δ*L*_3_ exhibited similar changes in both groups. The samples became brighter after four bleaching sessions and this parameter increased, but after the fifth session, a decrease in brightness was observed. This finding may be explained by the morphological effects of carbamide peroxide gel on dentin during the bleaching process [44]. Carbamide peroxide creates an acid etching appearance in dentin, increases the diameter of dentinal tubules, causes demineralization of dentin and noticeable changes in its inorganic composition [45], and increases the permeability of dentin tubules [44] during the bleaching process.

According to the research by Santos et al. [45], it seems that the carrier gel of carbamide peroxide also has a detrimental effect on dentinal structure.

Perhaps, the increase in brightness up to the fourth session can be attributed to the surface changes that cause more penetration of the bleaching material in the dentin. On the other hand, the cumulative effect of dentin buffering after 4 bleaching sessions may prevent further penetration of the bleaching agent into the dentinal tubules. In addition, MTA discoloration has been reported after exposure to bleaching agents [20], which could cause a decrease in the brightness of the samples after 5 sessions of bleaching.

Parameter Δ*b*_3_ shows the same pattern of changes during 5 sessions of bleaching in both groups. A decrease in yellowness and color shift to blue resulted in the improvement of tooth color observed during the sessions. This finding had the most effect on patient satisfaction [46].

According to Δ*E*_3_ (MTA-stained and bleaching sessions), the number of bleaching sessions to reach the threshold of ∆*E* < 3.3 was lower in group A than in group B (2 vs. 2.8) and more samples (8 vs. 5) reached to ∆*E* < 3.3 in group A. In addition, Δ*E*_4_ (intact teeth and bleaching sessions) showed 4 teeth from group A and 1 tooth from group B reached to ∆*E* < 3.3.

According to the results of this study, the mean numeral values of Δ*E*_3_ during bleaching sessions were higher in group B compared with group A. Acidic condition of bleaching agents causes more calcium and bismuth to be released from MTA and more Si ions to be deposited on its surface [20]. Therefore, it seems that after carbamide peroxide application in group B, more release of bismuth from MTA may be the cause of higher Δ*E*_3_ values in this group.

The threshold of perceptible and acceptable color change (∆*E*) is various [47] depending on the type of material used in different studies and who judges it. In the present study, OrthoMTA was tested. It is recommended that other types of materials used in regenerative treatments be reviewed as well.

## 5. Conclusion

Based on this study, MTA removal before bleaching treatment may be helpful to reduce the number of bleaching sessions and its harms. Although, the partial removal of MTA did not have a considerable effect in improving the tooth discoloration.

## Figures and Tables

**Table 1:** Color assessment stages.

*T*_0_	Intact teeth
*T*_1_	MTA-stained teeth
*T*_2_	Before MTA removal (in group A)
*T*_3_	After MTA removal (in group A)
T_4_−*T*_*B*_	After each bleaching session

**Table 2:** The stages of ∆*E*, ∆*L*, and ∆*b* calculation.

Δ*E*_1_	Intact teeth and MTA-stained (*T*_0_/*T*_1_)
Δ*L*_1_	
Δ*b*_1_	
Δ*E*_2_	Before and after MTA removal (*T*_2_/*T*_3_) (in group A)
Δ*L*_2_	
Δ*b*_2_	
Δ*E*_3_	MTA-stained teeth and bleaching sessions (*T*_1_/*T*_4_–*T*_8_)
Δ*L*_3_	
Δ*b*_3_	
Δ*E*_4_	Intact teeth and bleaching sessions (*T*_0_/*T*_4_–*T*_8_)
Δ*L*_4_	
Δ*b*_4_	

## Data Availability

The data used to support the findings of this study are available from the corresponding author upon request.
